# 109 Acceptability of Antiviral Prophylaxis in a VA Post-Acute and Long-Term Care Setting

**DOI:** 10.1017/ash.2026.10526

**Published:** 2026-06-23

**Authors:** Christopher Ly, Lyna Lam, Alexander Mink, Josephine Gresko, Jihye Kim, Kimberly Lee, Sangeeta Sastry

**Affiliations:** 1 VCU Health; 2 VCU Medical Center; 3 Virginia Commonwealth University

## Abstract

**Background:** Antibiotic allergy labels are common in clinical practice but rarely represent true IgE-mediated allergies. Mislabeling leads to increased utilization of broad-spectrum antibiotics, rates of Clostridioides difficile infection, drug toxicities, and healthcare costs. National societies recommend systematic confirmation of patient-reported antibiotic allergies as a core antibiotic stewardship activity, but risk-stratification tools remain under-utilized. We sought to assess the perceptions of antibiotic allergies in hospital passersby and stratify those at risk of true antibiotic allergy to encourage discussions on de-labeling or additional testing with appropriate healthcare providers and to ultimately optimize antibiotic use. **Methods:** During World Antibiotic Awareness Week 2025, a RedCap survey was offered to hospital passersby at Virginia Commonwealth University Medical Center over a 4-hour period. Surveys were completed on electronic tablets in the presence of healthcare providers. Validated decision tools (PEN-FAST, CEPH-FAST, and SULF-FAST) were used to stratify patients based on their risk of true antibiotic allergies. Scores of 0 indicate very low (<1%) risk of a positive antibiotic allergy test; scores of 1–2 indicate low (5%) risk; and scores of 3 indicate moderate (20%) risk. Responses were analyzed using descriptive statistics. **Result:** Eighty-four participants, spanning 8 to 81 years old and 68% of them female, completed the RedCap survey. Of those, 48 (57.1%) unique participants reported at least one allergy. The most common allergy reported was penicillin (34/48), followed by sulfa drugs (19/48) and cephalosporins (12/48). Of the 48 participants, 12/48 (25%) scored 0 on all applicable risk-stratification tests, indicating a very low risk for allergy. They were encouraged to discuss prompt antibiotic allergy de-labeling with their primary care provider following a risk-benefit discussion. A total of 18/48 (37.5%) participants on all applicable risk stratification tests scored 1 or 2, indicating a low risk for allergy. They were encouraged to seek referral to a specialist for allergy testing and likely antibiotic allergy de-labeling. The remaining participants who scored 3 or more indicating moderate to severe risk were not recommended for antibiotic allergy de-labeling. **Conclusion:** In passersby of a large tertiary care hospital, our findings showed a high proportion (30/48, 62.5%) of those self-reporting antibiotic allergies are at very-low- to low-risk for a true IgE-mediated allergy. This suggests an opportunity for methodical confirmation and implementation of antibiotic risk stratification in healthcare settings to ultimately improve stewardship of our antibiotic allergy labels. Healthcare providers must be empowered to become frontline stewards of antibiotic allergy labels to optimize prescribing patterns, promote appropriate antibiotic use, and ultimately improve patient care.

